# Pregnant Women’s Perception and Knowledge of the Impact of Obesity on Prenatal Outcomes—A Cross-Sectional Study

**DOI:** 10.3390/nu15112420

**Published:** 2023-05-23

**Authors:** Howaida Khair, Mo’ath F. Bataineh, Kornelia Zaręba, Shamsa Alawar, Sara Maki, Gehan Sayed Sallam, Afra Abdalla, Sharon Mutare, Habiba I. Ali

**Affiliations:** 1 Department of Obstetrics and Gynecology, College of Medicine and Health Sciences, United Arab Emirates University, Al Ain 17666, United Arab Emirates; 2 Department of Nutrition and Health, College of Medicine and Health Sciences, United Arab Emirates University, Al Ain 17666, United Arab Emirates; 3 Department of Veterinary Medicine, College of Agriculture and Veterinary Medicine, United Arab Emirates University, Al Ain 15551, United Arab Emirates

**Keywords:** obesity, body mass index, BMI, gestational weight gain, GWG, pregnancy, perception, pregnancy knowledge, pregnancy complications

## Abstract

The prevalence of obesity and overweight has been rapidly increasing and is significantly higher among adult females in the Arab States. The aim of the present study was to explore pregnant Emirati women’s perception of their weight, their knowledge of the healthy gestational weight gain, and the possible weight-related pregnancy complications. A total of 526 self-administered questionnaires were obtained with a response rate of 72%. The majority (81.8%, *n* = 429) entered pregnancy as overweight or obese. The percentage of pregnant women who underestimated their weight category was 12.1% in normal weight participants, 48.9% in overweight participants, and 73.5% in obese participants (*p* < 0.001). The overweight and obese participants were 13 times more likely to underestimate their weight status and 3.6 times more likely to correctly select their healthy gestational weight gain. Women’s awareness of pregnancy-related complications due to weight varied from 80.3% for diabetes to 44.5% for fetal complications; their awareness of breastfeeding difficulty was the lowest at 2.5%. Moreover, there was a misconception about personal BMI and the appropriate range for gestational weight gain (GWG). Healthy lifestyle counselling urgently needs to be addressed in preventative health programs such as pre-marital and preconception counselling.

## 1. Introduction

The prevalence of overweight and obesity has nearly tripled worldwide since 1975 and poses a significant public health threat increasing the risk of non-communicable chronic diseases worldwide [1]. In the Arab States of the Gulf Cooperation Council (which includes Bahrain, Kuwait, Oman, Qatar, Saudi Arabia, and the United Arab Emirates), obesity and overweight prevalence are among the highest worldwide [2]. It is significantly higher in adult females in Kuwait (44%), Qatar (42%), and Saudi Arabia (41%) [2]. A progressive increase in the prevalence of obesity and overweight in the general population has been observed in the United Arab Emirates (UAS) since 1989, reaching 31.7% in 2016 and rising more recently to 66.4% [3,4]. The rates were slightly higher in women compared to men, with 69.4% for women and 65.4% for men [4]. The high levels of obesity and overweight reflect rapid economic growth and changes in dietary habits and lifestyle [3]. These changes have resulted in less consumption of healthier food and a shift in physical activity levels from the traditional, physically active lifestyle to a more modern, sedentary indoor lifestyle in the UAE [5].

Overweight and obesity are defined as abnormal or excessive fat accumulation that may impair health [6]. They are classified using the body mass index (BMI), defined as a person’s weight in kilograms divided by the square of his height in meters (kg/m^2^) [1]. In 2009, the Institute of Medicine (IOM) and the National Research Council issued the document “Weight Gain During Pregnancy: Reexamining the Guidelines”. These guidelines were formulated as a range for healthy gestational weight gain (GWG) for each category of pre-pregnancy BMI. Based on pre-pregnancy BMI, the total GWG range in kg is determined as underweight (<18.5 kg/m2):12.5–18, normal weight (18.5–24.9 kg/m2): 11.5–16, overweight (25.0–29.9 kg/m2): 7–11.5, and obese (≥30.0 kg/m2): 5–9 [7].

There is good evidence from extensive cohort studies and meta-analyses that maternal obesity and overweight are associated with several adverse pregnancy outcomes in mothers and their babies [8,9,10,11]. There is a significant increase in maternal complications, including developing gestational diabetes mellitus (GDM), gestational hypertensive disorders (GHp), pre-eclampsia, and requiring operative deliveries [4,8,9,12,13,14,15]. The recommended weight gain ranges are those that are mostly consistent with good outcomes, including reduced postpartum weight retention, reduced maternal obesity and non-excessive GWG. A GWG above IOM recommendations significantly increases the risk of postpartum weight retention up to 1 year after delivery [16]. Moreover, it significantly impacts delivery and postpartum complications such as childhood obesity and shoulder dystocia [11,16]. For the fetus, neonatal adverse outcomes include macrosomia, being large for gestational age (LGA), fetal defects, congenital anomalies, and perinatal death [4,8,9,12,13,14,15].

Several studies of women from different ethnicities, ages, and education levels revealed that women had misperceptions regarding their weight, and they lack knowledge about the appropriate GWG [15,17]. Moreover, when the measured BMI was compared to the self-assigned one for the assessment of obesity among adult participants, there was tendency to overestimate the height and underestimate the weight [18]. Haakstad et al. revealed that women with a BMI ≥ 25 may have difficulty with accurate self-perception as overweight or obese [19]. In a systematic study, which included 173,971 study participants, the self-reported BMI was often inaccurate, with underestimates being more common than overestimates [20].

Previous studies suggest that the major issue is inadequate antenatal education by healthcare providers regarding healthy GWG [21,22,23]. Deputy et al. revealed that only 26.3% of women reported receiving healthcare provider advice consistent with the 2009 IOM recommendations, whereas 15.5% received advice that were below recommendations and 26.0% did not receive advice at all [21].

However, to the best of our knowledge, there is a lack of research from the UAE exploring women’s knowledge and perception of obesity, and its potential adverse effects on pregnancy outcomes. Moreover, there are no available studies of the level of antenatal education provided by healthcare providers during pregnancy about a healthy diet and lifestyle. This study, therefore, aimed to describe Emirati women’s perception and knowledge of their pre-pregnancy weight depending on age, parity, educational level, and BMI. Their awareness and the level of information provided by healthcare providers regarding the appropriate weight gain during pregnancy, and their knowledge of obesity-related complications (maternal and infant) during pregnancy were also evaluated.

## 2. Materials and Methods

The study was conducted in accordance with the Declaration of Helsinki and approved by the Institutional Bioethics Committee of Al Ain Medical District Human Research Ethics Committee (IRR 533/17). Informed consent was obtained from all subjects involved in the study.

This study was conducted in Al Ain due to the high percentage (about 30%) of Emirati citizens. According to Emirates of Abu Dhabi birth statistics, in 2019, there were 37,160 live births (16,670 were citizens) in the Abu Dhabi Emirate, including 56.6% in the Abu Dhabi region, followed by the Al Ain region with 40.0%, and the Al Dhafra region with 2.7% [24] (UAE Birth statistics 2019). The study population consisted of randomly selected pregnant Emirati women attending the selected prenatal clinics at Tawam and Al Ain hospitals during the study period. Their gestational age ranged from 24 to 35 weeks. Each of the two hospitals has approximately 5000 deliveries per annum. Informed consent was obtained from each participant before the interview.

The study was conducted between 1 September 2018 and 31 November 2019. During the study period, a total of 730 women were approached to participate, 526 questionnaires were returned, and the response rate was 72%.

### 2.1. Methods

The pregnant women were asked to complete an anonymous survey. Information on maternal characteristics was collected in the clinic on the same day of the interview by the research assistants with the help of trained nurses. Specifically, maternal age, educational level, gravidity, parity, and the number of miscarriages or ectopic pregnancies were recorded. Weight and height were measured on the day of the interview according to antenatal care guidelines by the National Institute of Clinical Excellence 2021. The pre-pregnancy weight was obtained from the electronic health information system within six months of the index pregnancy to calculate their pre-pregnancy BMI and correctly categorize their weight status.

Minimum sample size for the current study was determined using G*Power software (version 3.1.9.7) for the chi-square analysis with four categories. The calculation revealed the need for a minimum sample size composed of 281 participants to perceive a medium effect size of 0.30 with a significance level set at α =  0.05 and power = 0.99. A total of 526 pregnant women participated in the study.

The data collection instrument was developed by two obstetricians and reviewed by nutrition experts from the UAEU and clinical nurses. Considering their suggestions and recommendations, the tool was modified. In addition, the questionnaire was translated into Arabic by a local language expert.

### 2.2. Questionnaire

The understanding of the questionnaire was pilot tested by 10 lay women. The phrasing of the questions was edited according to the women’s feedback. The questionnaire had three sections. The first part of the questionnaire included basic sociodemographic information, parity, gravidity and miscarriage or ectopic pregnancies, and anthropometric measurements. Age was reported and then cross-checked with the date of birth. The second part of the questionnaire was developed to assess the accuracy of the women’s BMI perception and their knowledge regarding risks associated with overweight and obesity before and during gestation. More specifically, women were asked to identify their pre-pregnancy weight status as underweight, normal weight, overweight, or very overweight/obese and, based on their responses, what they thought was the appropriate pregnancy weight gain for themselves. A possible response of “I do not know” was also provided. Women were then asked if they believed obesity in pregnancy or excess GWG was related to various maternal and infant risks and complications. The correct response was based on the recommendations for weight gain by the IOM issued in 2009. Women who replied positively were asked to identify these risks and complications from a provided list of 5 possible maternal outcomes and 5 neonatal complications (Supplementary Materials File). Lastly, in the third section, women were asked to report if they had received, from their healthcare providers (physicians or nurses), verbal or written information with regards to lifestyle (exercise and weight management) and nutrition (healthy foods, amount to consume, and foods and drinks to avoid).

### 2.3. Analysis

Women were categorized based on whether they correctly identified the GWG recommendation and on pre-pregnancy weight status. Weight status was defined based on well-accepted pre-pregnancy BMI levels: underweight, BMI < 18.5 kg/m^2^; normal weight, 18.5 ≥ BMI > 25.0 kg/m^2^; overweight, 25 ≤ BMI < 30 kg/m^2^; and obese, BMI ≥ 30 kg/m^2^.

### 2.4. Statistical Methods

All statistical analyses were performed using the SPSS version 28.0 statistical package (IBM Corporation, Chicago, IL, USA). Data normality was tested with the use of the Kolmogorov–Smirnov test. All data were presented using frequency and percentages for the categorical variables and mean and standard deviation for the continuous variables. Statistical tests were carried out with the use of two-sided tests to detect significance. A chi-square analysis was performed to detect association between categorical variables. An independent samples Kruskal–Wallis test was conducted to detect differences in perception and knowledge based on BMI classification.

Furthermore, two logistic regression tests were carried out to investigate the impact of confounding factors on the BMI classification during pregnancy and the correct healthy gestational weight gain as outcome variables. The logistic regression used a binary coding of 0 for the wrong perception and 1 for the correct perception about the BMI classification during pregnancy and the healthy gestational weight gain. The confounding factors selected for the final model included age, pre-pregnancy BMI, educational level, and parity. Both age and pre-pregnancy BMI as confounding factors included in the logistic regression tests were selected with the use of a univariate general linear model, with a cut-off value of *p*  <  0.20 to be included in the final model. In addition, parity and education were included in the final analysis due to the hypothesis that women with previous pregnancies or women with higher levels of education might have received knowledge about obesity’s negative influence on perinatal outcomes. For the final analysis, and due to the low number of underweight participants, the BMI classification was categorized into two categories: category 1 representing underweight and normal and category 2 representing overweight and obese. Statistical significance was obtained with a *p*-value < 0.05.

## 3. Results

### 3.1. Study Group Characteristics

The average maternal age of participants was 31.5 ± 5.9 years, and the average pre-pregnancy calculated BMI was 30.1 ± 5.6 kg/m^2^ (Table 1). Overall, most participants are in the age group 30 to 35 years old (32.5%), are obese (45.9%), have university degrees (54.4%), have given birth more than once (65.8%), and have not suffered miscarriage or ectopic pregnancy (56.8%) (Table 1).

### 3.2. Perception of the Weight

**Figure 1:**
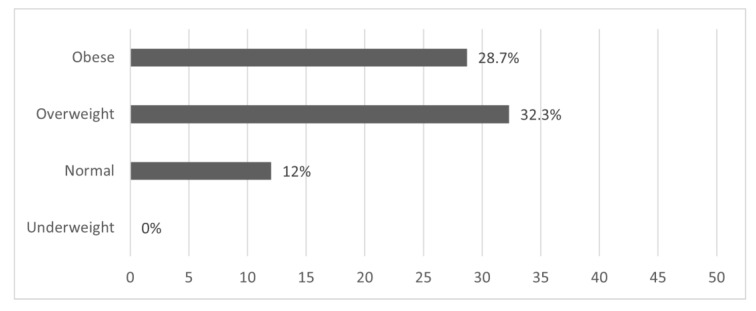
Percentage of pregnant women who correctly chose recommended gestational weight gain based on their BMI classification (*n* = 526).

To assess the association between BMI classification and underestimation of weight category, pregnant women with underweight status and missing value cases were excluded before a chi-square analysis was performed (Figure 1). The percentage of pregnant women who underestimated their weight category was 12.1% in normal weight participants, 48.9% in overweight participants, and 73.5% in obese participants (χ^2^ (2) = 102.734; *n* = 511; *p* < 0.001). Furthermore, an independent samples Kruskal–Wallis test showed significant differences in underestimation of weight category between the three BMI classifications (χ^2^ (2) = 102.533; *n* = 511; *p* < 0.001), with a mean rank in underestimation of weight category score of 306.37 for the obese, 243.44 for the overweight, and 149.38 for the normal weight. Obese participants were significantly different from both overweight (*p* < 0.001) and normal weight (*p* < 0.001) participants; furthermore, overweight participants were significantly different from normal weight participants (*p* < 0.001).

### 3.3. Knowledge about Recommended Weight and Weight Gain during Pregnancy

Assessment of the study participants’ knowledge about the recommended weight gain during pregnancy showed that the total percentage of participants who correctly selected the weight gain suitable for their pre-pregnancy weight category was 26.8% (*n* = 141). A chi-square analysis was conducted to investigate the association between the pre-pregnancy weight category and knowledge about recommended weight gain during pregnancy. The percentage of pregnant women who correctly chose the suitable weight gain during pregnancy was 0.0% in underweight participants, 12.0% in normal weight participants, 32.3% in overweight participants, and 28.7% in obese participants (χ^2^ (6) = 21.346; *p* = 0.002) (Figure 1). Furthermore, an independent samples Kruskal–Wallis test showed significant differences in correctly selecting the recommended weight gain during pregnancy between the different BMI classifications (χ^2^ (3) = 14.758; *p* = 0.002), with a mean rank score in correctly choosing weight gain during pregnancy of 277.88 for the overweight, 268.61 for the obese, 224.45 for the normal weight, and 193.00 for the underweight. Overweight participants were significantly different from normal weight (*p* < 0.001) participants. Obese participants were significantly different from normal weight participants (*p* = 0.002). No significant differences were detected between overweight and obese, overweight and underweight, obese and underweight, or normal and underweight.

### 3.4. Awareness of Obesity-Related Complications

A majority of 432 participants (82.1%) were aware that extra weight before and during pregnancy increases the risk of pregnancy-related complications for the mother (Table 2). Perception about pregnancy-related complications for the mother varied from as high as 80.3% for diabetes to as low as 2.5% for difficulty in breast feeding. Except for diabetes (χ^2^ = 13.576; *p* = 0.004), all perceived pregnancy-related complications for the mother were not associated with the pre-pregnancy weight category (*p* > 0.05).

Few participants (*n* = 234, 44.5%) were aware that the extra weight was associated with increased risk of pregnancy-related complications for the fetus. Perceptions about pregnancy-related complications for the fetus varied from as high as 70.5% for birth trauma to as low as 3.4% for birth defects. No statistical associations between pregnancy-related complications for the fetus and different pre-pregnancy weight categories were detected.

### 3.5. Association of Underestimated BMI, or Healthy GWG Awareness

A Spearman’s rho correlation analysis revealed a significant, weak correlation between underestimation of weight status and correctly selecting the healthy gestational weight gain (r = 0.153; *p* < 0.001). The logistic regression analysis results show that only the pre-pregnancy weight category was statistically associated with both outcome variables (*p* < 0.001) (Table 3). The overweight and obese participants were 13.335 times more likely to underestimate their weight status and 3.597 times more likely to correctly select their healthy gestational weight gain. No significant association with the outcome variables was detected for age group, parity, and educational level.

### 3.6. Information Provided by Healthcare Providers

**Figure 2:**
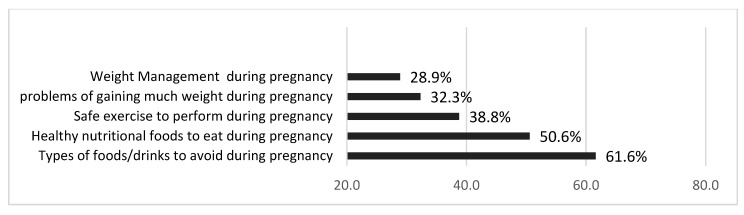
Information provided by healthcare providers in percentages (*n* = 526).

When asked about eating habits, except for healthy nutritional foods (50.6%) and foods and drinks to avoid during pregnancy (61.6%), a minority of participants indicated that they received information about weight management, safe exercises and amount of exercise, and nutritional foods to eat during pregnancy (Figure 2).

## 4. Discussion

The majority (81.8%, *n* = 429) entered pregnancy as overweight or obese. The overweight and obese participants were over 13 times more likely to underestimate their weight status and 3.6 times more likely to correctly select their healthy gestational weight gain. The percentage of pregnant women who correctly chose the suitable weight gain during pregnancy was 0.0% in underweight participants, 12.0% in normal weight participants, 32.3% in overweight participants, and 28.7% in obese participants. Perception about pregnancy-related complications for the mother connected with obesity varied from as high as 80.3% for diabetes to as low as 2.5% for difficulty in breast feeding. Few participants (44.5%) were aware that the extra weight was associated with an increased risk of pregnancy-related complications for the fetus.

Obesity and overweight pose a significant public health threat increasing in prevalence in the Middle East. In the present study, 429 (81.8%) participants contemplated pregnancy either as overweight or obese. According to Fayed at al. the percentage of obesity (34.8%) and overweight (33.3%) in pregnancy in Saudi Arabia was also very high; however, it was lower than in the present study [25].

The inaccurate perception of their own weight and BMI was also common among pregnant women in other studies [26,27,28]. Australian and Canadian researchers revealed that only 30% of Australian and 10% of Canadian obese pregnant women accurately classified their BMI [26,27]. Moreover, among Saudi pregnant women only 24% of the obese women accurately identified their weight category as obese [28].

A study that assessed Australian pregnant women’s knowledge relating to GWG confirms a notable lack of maternal knowledge about guidelines of appropriate GWG [14]. In the present study, the association of social and demographic independent factors with the correct perceptions of BMI classification and healthy GWG revealed that only BMI was significantly associated with correct perceptions of BMI classification and healthy GWG. Participants who were overweight or obese were less likely to correctly select the right BMI classification compared with underweight or normal participants. In contrast, overweight or obese participants were more likely to correctly select the right healthy weight gain during pregnancy compared with participants who were underweight or normal.

There is evidence that excessive and poor GWG is associated with adverse perinatal outcomes [11,29,30]. Pre-pregnancy high BMI and excessive GWG are associated with an increase in the risk of developing GDM, GHp, and macrosomia [1,13,31]. A systematic review and meta-analysis of around one million pregnant women showed there was an increased risk of small for gestational age and preterm birth with a GWG lower than the IOM recommendation. In contrast, women with excessive GWG were at increased risk of macrosomia, large for gestational age, and increased cesarean section delivery rate. A systematic review of cohort studies from Europe and North America estimated that 23.9% of pregnancy complications were attributable to maternal overweight/obesity, and 31.6% of large gestational-age infants were attributable to excessive gestational weight gain [31]. The participants in our study had shown a low rate of awareness of maternal and perinatal outcomes associated with obesity and overweight. Additionally, they were more aware of adverse perinatal outcomes compared to maternity-related health risks associated with obesity. Knowledge of the effect of obesity/excessive GWG on pregnancy outcomes was broadly investigated [9,11]. In the present study GDM was the most recognized adverse outcome by the participants, followed by post-partum maternal weight retention, difficult labor, hypertension, and cesarean section. A similar study reported that 94% of the women surveyed believed that excess GWG or obesity would be associated with increased pregnancy complications [28]. Moreover, our results show that the most recognized fetal and neonatal complications by participants were birth trauma, macrosomic babies and child obesity, followed by miscarriages and birth defects.

A healthy and balanced diet is important during a lifetime, particularly during pregnancy; pregnant women’s diet should maintain their own needs and the nutritional needs of their developing fetus and future breastfeeding practices. The American College of Obstetricians and Gynecologists recommends calculating pre-pregnancy BMI at the initial prenatal care visit and discussing gestational weight gain [32]. Antenatal education aims at improving the health of the mother, baby, and families. O’Brien et al. revealed that pregnant women who receive a low level of antenatal education are at an increased risk of excessive and inadequate GWG [15]. During pregnancy, women are more aware and motivated regarding the importance of healthy nutrition. They are also more receptive to information about what to eat and avoid [1,33]. Our results showed that the overall received prenatal education was low among participants. However, information received about healthy diets and healthy nutritional foods—to eat and to avoid—were higher than prenatal education received about weight management and safe exercise during pregnancy. It has been proven that implementing healthy habits in early pregnancy may reduce the frequency of gestational diabetes mellitus in overweight/obese pregnant women with no risk of preterm birth [9]. Thus, counselling plays an important role in improving women’s awareness about GDM risks related to the fetus and mother [34]. Women care mostly about fetal wellbeing during pregnancy; therefore, they will be more willing to strictly follow recommendations. Moreover, it is proven that obesity in pregnancy can transmit poor cardiometabolic health across generations [34]. Studies in animals suggest that adaptations connected with obesity that occur in fetal life can remodel the structures of major organs, including the brain, kidney, and pancreas [35,36].

### Strengths and Limitations of the Study

To the best of our knowledge, the present study represents the first research from the UAE exploring women’s knowledge and perception of obesity, and its potential adverse effects on pregnancy outcomes. Moreover, the study was conducted in a large group of females that might be representative for the country. It draws attention to the significantly high prevalence of obesity in females and low level of awareness and education in this field, which is a novel finding.

The study is preliminary data based on the present authors’ questionnaire, which may be subject to errors due to lack of validation. Nevertheless, the questionnaire was developed by clinicians, educators, and psychologists and includes many scientifically important pieces of information. The study was also performed in a specific group, i.e., in a sociocultural environment where one may find different lifestyles and eating habits. Moreover, the cross-sectional nature as well as the lack of follow-up that could reveal further weight gain are limitations of the study. Therefore, it seems reasonable to conduct an assessment of the long-term impact of obesity and obesity awareness on the future condition of mothers and children using validated questionnaires.

## 5. Conclusions

Obesity and excessive GWG are increasing in prevalence. The knowledge of the influence of obesity on the pregnancy outcome is very low. Moreover, there is a misconception about personal BMI and the appropriate range of GWG. Antenatal education including healthy lifestyles during pregnancy is urgently needed, and healthy lifestyle counselling should be incorporated in preventative health programs such as pre-marital and preconception counselling. Pregnant women’s access to information and education from a multifaceted program will empower women to take the lead for improvement toward a healthy pregnancy and life in general.

## Figures and Tables

**Table 1:** Sociodemographic data of the respondents (*n* = 526).

Factor	*n* (%)
Age (Years)	
18–23	49 (9.3)
24–29	157 (29.8)
30–35	171 (32.5)
≥36	149 (28.3)
Body Mass Index Classification	
Underweight	5 (1.0)
Normal	92 (17.5)
Overweight	189 (35.9)
Obese	240 (45.9)
Education	
High school or less	225 (42.8)
Technical diploma	11 (2.1)
University degree	286 (54.4)
Parity	
0	88 (16.7)
1	89 (16.9)
≥2	346 (65.8)
Missing	3 (0.6)
Miscarriage and Ectopic	
0	299 (56.8)
≥1	224 (42.6)
Missing	3 (0.6)

**Table 2:** Participants’ perceived effect of their weight category on maternal and fetal complications (*n* = 526).

Factor	Total (*n* = 526)	Weight Category				χ^2^ *	*p*-Value
		Underweight (*n* = 5)	Normal (*n* = 92)	Overweight (*n* = 189)	Obese (*n* = 240)		
**Too much weight gain in pregnancy or being overweight can increase problems in pregnancy for the mother?**							
No	39 (7.4)	0 (0.0)	7 (7.6)	13 (6.9)	19 (7.9)	2.508	0.868
Yes	432 (82.1)	5 (100)	76 (82.6)	159 (84.1)	192 (80.0)		
Not sure	55 (10.5)	0 (0.0)	9 (9.8)	17 (9.0)	29 (12.1)		
**What kind of problems to the mother?**							
Diabetes	347 (80.3)	1 (20.0)	61 (80.3)	124 (78.0)	161 (83.9)	13.576	0.004
Caesarean Section	91 (21.1)	0 (0.0)	13 (17.1)	32 (20.1)	46 (24.0)	3.102	0.376
Big Baby (Macrosomia)	154 (35.6)	1 (20.0)	28 (36.8)	56 (35.2)	69 (35.9)	0.601	0.896
Difficult Labor	197 (45.6)	1 (20.0)	38 (50.0)	63 (39.6)	95 (49.5)	5.369	0.147
Preterm Labor	43 (10.0)	0 (0.0)	6 (7.9)	17 (10.7)	20 (10.4)	1.055	0.788
Post-term Pregnancy	25 (5.8)	0 (0.0)	3 (3.9)	9 (5.7)	13 (6.8)	1.124	0.771
Post-partum Weight Retention	216 (50.0)	2 (40.0)	34 (44.7)	87 (54.7)	93 (48.4)	2.645	0.450
Difficulty in Breast Feeding	11 (2.5)	0 (0.0)	1 (1.3)	3 (1.9)	7 (3.6)	1.809	0.613
Miscarriage	16 (3.7)	0 (0.0)	1 (1.3)	3 (1.9)	12 (6.3)	6.369	0.095
Hypertension	133 (30.8)	0 (0.0)	20 (26.3)	48 (30.2)	65 (33.9)	3.811	0.283
**Too much weight gain in pregnancy or being overweight can increase problems in pregnancy for the fetus?**							
No	85 (16.2)	1 (20.0)	20 (21.7)	36 (19.0)	28 (11.7)	7.016	0.319
Yes	234 (44.5)	2 (40.0)	39 (42.4)	82 (43.4)	111 (46.3)		
Not sure	207 (39.4)	2 (40.0)	33 (35.9)	71 (37.6)	101 (42.1)		
**What kind of problems to the fetus?**							
Large Baby	141 (60.3)	0 (0.0)	24 (61.5)	52 (63.4)	65 (58.6)	3.534	0.316
Birth Trauma	165 (70.5)	1 (50.0)	25 (64.1)	61 (74.4)	78 (70.3)	1.772	0.621
Low Glucose Level	59 (25.2)	1 (50.0)	10 (25.6)	21 (25.6)	27 (24.3)	0.709	0.871
Jaundice	19 (8.1)	0 (0.0)	4 (10.3)	7 (8.5)	8 (7.2)	0.558	0.906
Childhood Obesity	49 (20.9)	0 (0.0)	10 (25.6)	18 (22.0)	21 (18.9)	1.375	0.711
Adulthood Obesity	25 (10.7)	0 (0.0)	5 (12.8)	8 (9.8)	12 (10.8)	0.502	0.919
Baby Death	19 (8.1)	0 (0.0)	2 (5.1)	7 (8.5)	10 (9.0)	0.781	0.854
Birth Defects	8 (3.4)	0 (0.0)	2 (5.1)	1 (1.2)	5 (4.5)	2.013	0.570
NICU Admission	23 (9.8)	0 (0.0)	1 (2.6)	12 (14.6)	10 (9.0)	4.761	0.190

* Chi-square test.

**Table 3:** Association of underestimated BMI or healthy GWG awareness with maternal age, pre-pregnancy weight status, educational level, and parity (*n* = 526).

Factor	Underestimated BMI Odds Ratio (CI 95%)	*p*-Value	Awareness of Healthy GWG Odds Ratio (CI 95%)	*p*-Value
**Age (Years)**		0.668		0.786
18–23	1		1	
24–29	0.714 (0.324–1.575)		0.769 (0.353–1.678)	
30–35	0.691 (0.298–1.600)		0.988 (0.433–2.258)	
≥36	0.866 (0.360–2.085)		0.870 (0.365–2.074)	
**Body Mass Index Classification**		<0.001		<0.001
Underweight/Normal	1		1	
Overweight/Obese	13.335 (6.857–25.935)		3.597 (1.842–7.023)	
**Education**		0.153		0.194
High school or less	1		1	
Technical diploma	0.334 (0.092–1.219)		1.146 (0.289–4.550)	
University degree	0.776 (0.525–1.149)		1.460 (0.969–2.200)	
**Parity**		0.898		0.742
0	1		1	
1	0.893 (0.450–1.772)		1.313 (0.652–2.644)	
≥2	0.863 (0.463–1.611)		1.134 (0.590–2.179)	

## Data Availability

Not applicable.

## Supplementary Materials

The following supporting information can be downloaded at: https://www.mdpi.com/article/10.3390/nu15112420/s1, Questionnaire.
