# Supplementary material for: Pregnant Women’s Perception and Knowledge of the Impact of Obesity on Prenatal Outcomes—A Cross-Sectional Study
Source: Nutrients. 2023 May 23;15(11):2420. doi: 10.3390/nu15112420 (PMC10254899; doi:10.3390/nu15112420)
Supplement: Supplementary file 1 [file nutrients-15-02420-s001.zip › nutrients-2217537-supplementary.pdf]

Questionnaire on Women's Perception of Obesity & weight Gain During Pregnancy (Page 1)

(Page 1 should be completed by the research assistant and from 2 to 4 by the participant)

Socio-demographic data

1-Patient study number: - ☐☐☐

2- File No.: - ☐☐☐☐☐☐☐☐☐☐☐☐

3-Study Centre: -
Al Ain Hospital ☐
Tawam Hospital ☐

4-Age: - ☐☐

5- Date of birth...................DD/MM/YY

6-Educational level: -

Illiterate/Elementary ☐ Lower secondary education ☐ Secondary education ☐

Technical education ☐ College/university education ☐ Post graduate study ☐

7. Maternal height, pre-pregnancy weight and BMI:

Pre-pregnancy weight (kg)......... 8. Length (cm): .... 9. Body mass index................

8. Gravidity …… Parity………+ (Miscarriage/Ectopic Pregnancy) ………….

Study #...........................File#..

Questionnaire on Women's Perception of Obesity & weight Gain During Pregnancy (Page 2)

Pre-pregnancy weight/ height category and healthy weight gain during pregnancy:

Please answer the below questions by indicating (x) on the box

1. Based on your height and weight before pregnancy, what do you consider yourself to be?

Very over weight/ Obese ☐ Over weight ☐ Healthy weight ☐

Less than average ☐ I don't know ☐

2. For your height and weight before pregnancy, which of the following range of weight gain (extra weight you gain during the whole duration of pregnancy) do you think is the healthy for you?

12.5-18 Kg ☐ 11.5-16.5 Kg ☐ 7-11.5 Kg ☐ 5-9Kg ☐ I don't know ☐

3. Do you think if the woman is overweight/obese or have too much weight gain during pregnancy can cause problems for her during pregnancy?

Yes, ☐ No ☐ I don't Know ☐

Study #............................File#...

Questionnaire on Women's Perception of Obesity & weight Gain During Pregnancy (Page 3)

Please answer question (4) ONLY if your answer to question (3) was YES

4. What kind of problems? You can tick more than one answer

More possibility of having diabetes (high blood sugar) during pregnancy ☐

More likely do have caesarean delivery ☐

More likely to have long labor and difficult delivery ☐

Difficulty to ger rid of the excess weight she gained while pregnant ☐

Hypertension (High blood pressure during pregnancy) ☐

5. Do you think being overweight/ obese or have too much weight gain during pregnancy can cause problems for the baby (while in the womb) and during delivery?

Yes, ☐ No ☐ I don't Know ☐

Please answer question (6) ONLY if your answer to question (5) was YES

6. What kind of problems your belief that the baby may have after birth?

More likely to have" Big Baby" baby weight 4 kg or more at birth ☐

Birth truma due to difficult delivery ☐

Baby may be obese during childhood (up to 10 years) ☐

Increased risk of miscarriage (pregnancy loss before 20 weeks of pregnancy ☐

The baby may be born with birth defects e.g. abnormal heart ☐

Study #..........................File#..

Questionnaire on Women's Perception of Obesity & weight Gain During Pregnancy (Page 4)

Information given by nurse/midwife and or doctor during pregnancy clinic visits:

In my clinic visits during the pregnancy the doctor/ nurse/ midwife have talked to me or provided me with written leaflets about:
about the following:

Problems of gaining too much weight during pregnancy	Yes ☐	No ☐
Healthy and nutritional foods to eat during pregnancy	Yes ☐	No ☐
Types of food and drinks I should avoid while being pregnant	Yes ☐	No ☐
Types of safe exercise to do during pregnancy	Yes ☐	☐
Managing my weight during pregnancy	Y ☐	No ☐

10. Do you have any comment or suggestion, please add it below?

....
....
....
....
....
....
....

Thank you for your participation

If you have any question please contact the main researcher
Dr. Howaida Khair (hkhair@uaeu.aca.ae)

Study #...........................File#...
